# Characterization of the Aroma Profile and Main Key Odorants of Espresso Coffee

**DOI:** 10.3390/molecules26133856

**Published:** 2021-06-24

**Authors:** Simone Angeloni, Ahmed M. Mustafa, Doaa Abouelenein, Laura Alessandroni, Laura Acquaticci, Franks Kamgang Nzekoue, Riccardo Petrelli, Gianni Sagratini, Sauro Vittori, Elisabetta Torregiani, Giovanni Caprioli

**Affiliations:** 1 School of Pharmacy, University of Camerino, via Sant Agostino 1, 62032 Camerino, Italy; simone.angeloni@unicam.it (S.A.); ahmed.mustafa@unicam.it (A.M.M.); doaa.abouelenein@unicam.it (D.A.); laura.alessandroni@unicam.it (L.A.); laura.acquaticci@unicam.it (L.A.); astride.kamgang@unicam.it (F.K.N.); gianni.sagratini@unicam.it (G.S.); sauro.vittori@unicam.it (S.V.); elisabetta.torregiani@unicam.it (E.T.); giovanni.caprioli@unicam.it (G.C.); 2 RICH—Research and Innovation Coffee Hub, via E. Betti 1, 62020 Belforte del Chienti, Italy; 3 Department of Pharmacognosy, Faculty of Pharmacy, Zagazig University, Zagazig 44519, Egypt

**Keywords:** espresso coffee, aroma compounds, key-odorants, espresso coffee machine, SPME-GC-MS, GC-O

## Abstract

Espresso coffee (EC) is a common coffee preparation technique that nowadays is broadly widespread all over the globe. Its popularity is in part attributed to the intense aroma and pleasant flavor. Many researchers have studied and reviewed the aroma of the coffee, but there is a lack of specific review focused on EC aroma profile even if it is intensively investigated. Thus, the objective of the current review was to summarize the aroma profile of EC and how different preparation variables can affect EC flavor. Moreover, a collection of diverse analytical procedures for volatile analysis was also reported. The findings of this survey showed that the volatile fraction of EC is extremely complex, but just some compounds are responsible for the characteristic aroma of the coffee, such as some aldehyde, ketones, furanones, furans, sulfur compounds, pyrazines, etc. In addition, during preparation, some variables, e.g., temperature and pressure of water, granulometry of the coffee particle, and brew ratio, can also modify the aroma profile of this beverage, and therefore its quality. A better understanding of the aroma fraction of EC and how the preparation variables should be adjusted according to desired EC would assist coffee workers in obtaining a higher quality product.

## 1. Introduction

Coffee is a daily part of our food habits and our social life. Drinking habits, together with authentic processing and preparation methods, represent and characterize a typical coffee culture. Nowadays, with a more and more increasing demand for high-quality food products, coffee should be considered, and sometimes it is considered, a high-quality artisanal food such as wine [1]. Two species, *Coffea arabica* L. (arabica) and *Coffea canephora* Pierre ex A. Froehner (robusta), are responsible for 99% of global coffee production [2]. Depending on geographic, cultural, and social context, as well as on personal preferences, numerous preparation methods and extraction processes have been developed and then introduced in society since the discovery of coffee as a beverage [3]. One of the most common preparation techniques of southern Europe, Central America, and other areas is espresso coffee (EC). EC is a beverage prepared on client request, as the *espresso* word suggests, using hot water and high pressure aimed to rapidly obtained a concentrated short drink. This beverage is appreciated and loved perhaps for its unique sensorial properties, which include a heavy body and intense aroma, a bitter/acid taste, and a pleasant lingering aftertaste [4,5,6]. The coffee flavor is extremely complex as it contains more than 1000 compounds that affect the sensorial perceptions of the oral and nasal mucosa. The coffee chemical composition and then the relative sensorial perception that this beverage generates is related to several factors such as the characteristics of the land on which the crop was cultivated, the climate during the growing season, the care with which it was harvested, the processing methods adopted, the roasting process, the blending, the grinding, the preservation technique, and the brewing [7]. In the case of espresso coffee, the barista can play and adjust several brewing variables such as amount of roasted and ground (R&G) coffee, R&G coffee granulometry, the design of some machine tools (e.g., filter basket, perforated disk), tamping force, and water temperature and pressure, in order to obtain the desired coffee cup [7,8,9,10,11].

### 1.1. History 

How the “coffee” word is born and become popular is still not clear. The early forms of the word in English (coffee), in French (café), in German (kaffe), and in Italian (caffè) indicate a derivation from Arabic and/or Turkish [12]. Various theories were formulated, but no written evidence was found on who and where people have started to use this word. Often, the word dated back to Arabic and other times to the African language. In the 15th Century, the coffee fruits and plants were called the Arabic word “bunn” (bun) while, nowadays, in Arabic, coffee is named “kahva”. The word “kaffa” is probably an adulterated version of the word “kahva”. Moreover, Kaffa is also a province on the southwestern side of Ethiopia, which is considered the motherland of coffee plants. For this reason, coffee was named “kaffa” from the African language. The word “coffee” (kahve) in Turkish is used for the drink that is brewed by boiling this plant’s beans [13]. Over the ages, numerous legends and stories were narrated about the origin and discovery of coffee. The most reported is about a shepherd named Khaldi or Kaldi who noticed that his goats, including the oldest one, were frenetic; in other words, they ran and gamboled. Looking at this, he realized that his goats were acting in this strange way after eating red berries from a shrub. These red berries were coffee fruits, the fleshy drupes that covered the coffee seeds [13,14]. Knowing the origin and exactly the date of this event is not simple, but it is known that the wild coffee plant (*Coffea arabica*) originated from Ethiopia, where it was discovered in about AD 850 [15]. How, when, and by whom the coffee was exported to Arabia is not clear, but it is established that the first country to which coffee was taken for cultivation from Ethiopia was Yemen (old Arabia Felix). From Yemen, later on, coffee was exported to India (1600), Sri Lanka (Ceylon; 1600–1696), Java (1690 and 1699), and Réunion (Bourbon; 1715–1718). Around the middle of the 15th century, Arabians started to consumed coffee as a drink, and it was later introduced to other countries at different times such as Rome in 1625, France in 1644/1671, Oxford in 1650, London in 1652, Marseilles in 1659, Amsterdam in 1663, Paris in 1675, Hamburg in 1679, etc. The coffee arrival in Europe is attributed to a Dutch trader in the early 17th century, and at the beginning of the 18th century, the first plants were shipped to the Amsterdam Botanic Gardens, which later produced seeds and seedlings that were distributed to several botanical gardens in Europe [15,16]. Coffee was first introduced in Italy in the second half of the 17th century, probably in Venice, but the espresso coffee preparation was invented several years later. At that time, coffee was prepared with infusion methods, as commonly took place in Europe. In the 19th century, at-home coffee started to be prepared by a filtering method using a pot named *napoletana* or *cuccumella*. The increasing demand to consume this beverage outside the home pushed the development of a more efficient, faster, and cheaper method to brew a single cup of coffee for customers. Around Europe, engineering started to produce brewing machines in which the coffee could be stored and then decanted into individual cups when required. The first machine that employed pressure was a design by Angelo Moriondo of Turin in 1885. This machine used steam pressure to drive hot water into a compressed coffee cake. In 1901, Luigi Bezzera of Milan first invented the machine with multiple group heads that permitted brewing coffee at customers’ requests (express preparation). This patent was bought by the manufacturer Desiderio Pavoni, whose *Ideale* machine is considered the first espresso coffee machine to enter into commercial production [17]. Another important invention was by Achille Gaggia in 1947, who registered a patent for a lever-operated piston for driving water directly from the boiler to the coffee cake. The use of this system permitted the generation of higher water pressure, causing a crema on the surface of the drink for the first time. That beverage was named *crema caffè* (cream coffee). In 1961, Ernesto Valente invented the first semi-automatic espresso coffee machine (Faema E61). This was able to prepare a cup of coffee after another without waiting for the reheating of the boiler each time (continuous erogation). In Switzerland, the first automatic machine was designed by using micro-chip technology. This permitted to automatically adjust the temperature, the pressure, and the duration of the extraction. Later, this machine was implemented with the integration of the grinder and the so called super-automatic or bean to cup machine was born [7,18]. 

### 1.2. Main Coffee Species and Uses

The coffee plants belong to the *Rubiaceae* family, genus *Coffea*. More than 100 species are part of the *Coffea* genus [19], but only two, i.e., *Coffea arabica* L. (arabica) and *Coffea canephora* Pierre ex A. Froehner (robusta), are responsible for the majority of global coffee production [2]. *Coffea liberica* Hiern, originally found in Liberia, is the third used and commercialized coffee species, but it accounts only for less than 1% of the global coffee production [5,16]. Probably, the motherland of the *Coffea* genus is the humid tropical regions of Africa and islands in the West Indian Ocean. Originally, arabica was a shrub growing at an elevation between 1300 and 2000 m in the forests of the southwest of Ethiopia and north of Kenya, while robusta originated from the humid lowland forests of tropical Africa [15]. Coffee is an evergreen and perennial plant that possess a prominent vertical stem with a shallow root system. The feeder roots of robusta are concentrated very close to the soil surface, whereas those of arabica penetrate relatively deeper into the soil. On suckers, coffee leaves are opposite decussate, dark green, wavy, and shiny. They are characterized by conspicuous veins; the inflorescence is a condensed cymose type subtended by bracts. Coffee is a short-day plant; hence, the floral initiation takes place in short-day conditions of 8-11 h of daylight. Pollination takes place within 6 h after flowering. The fruit is a drupe, called cherry or berry, usually fleshy, generally containing two seeds (coffee beans) but sometimes only one, and in that case, the fruit assumes a rounded shape and is known as pea-berry [20]. It varies in size but very little in shape. Its color varies from yellow to black, though it is mostly orange to red. Seeds are elliptical or egg-shaped, and the seed coat is represented by the silverskin [16,21].

The first use of coffee began several hundreds of years ago in Africa, the native country of the wild plant. Here, the aborigines used different parts of the plants, i.e., leaves, fruits, and seeds, as food. In Ethiopia, dried berries were chewed to overcome the fatigue, and a mixture of R&G coffee with fat or oil was carried into the desert or long safaris as a source of nutrient food. Coffee fruit is also cooked with butter to make a salted flat cake, or the green coffee is roasted, ground, and then mixed with butter. In Africa, coffee was used in the ceremonies since sharing a cup of coffee meant exchange news, well-wishing with friends and relatives, and expressing respect to guests [16]. Today, coffee is commonly used for the preparation of cookies, snakes, and desserts [22]. For example, an iconic Italian dish is *Tiramisù* [23], or it is employed in spiritual preparation. Certainly, the major use of coffee is for beverage production, which is especially consumed as part of meals, during breakfast, during working breaks, during socialization with friends and relatives, for car drivers to avoid falling asleep, and for airline pilots to combat fatigue [22]. It can be consumed as it is or by adding milk, sugar, chocolate, spices, and alcoholic beverages. Coffee shops, which are widespread in the cities of almost every country, serve different kinds of coffee beverages such as americano, cappuccino, espresso, macchiato, caffè latte, decaf, mocha, Italian roast, medium roast, breakfast, latte, etc. [24]. Very common milk-based drinks are *cappuccino* and *caffè latte*. These preparations account for 90% of coffee consumption in the Anglophone world [7]. Coffee beverages can be prepared by several different methods and a huge variety of preparations, such as Turkish coffee, drip coffee, French press, espresso coffee, Moka pot, cold brew, etc., and can be characterized by extraction tools but can also be grouped by various key parameters influencing the final flavor profile, such as pressure, brewing time, compaction, etc. [15]. Turkish coffee is prepared by grinding beans to fine particles and then adding water and coffee powder in a pot (e.g., cesve). The water is brought to boil for no more than an instant, and then the heating is usually stopped. This generates a sturdy drink characterized by a foam layer on the surface and sediments that settle on the bottom of the cup. Drip coffee, also called filter coffee or pour-over brew, consists of brewing coffee by pouring hot water onto the coffee powder, generally milled as coarse particles. The water passes into the coffee by gravity, and the powder remains in a holder containing a filtering device. Different filter devices constituted of various materials, sizes, and shapes, are commercially available. The water can be poured onto coffee through the automatic system or manually. This method produces a coffee milder than Turkish’s one and with enhanced acidity and flavor. Rather coarse particles are used for French press as well. In this preparation technique, the water and R&G coffee are placed into a vessel known as French press, *cafetière*, coffee press, or coffee plunger, and the steeping is performed for a specific time (2–5 min), depending on the intensity of extraction the barista prefers. The liquid part is separated from the solid phase by using a plunger containing a filter device. Because of inefficient metal-mesh filtration, higher content of sediments is usually attained compared to drip coffee. The most popular household appliance for brewing coffee in Italy is the Moka pot. This tool is composed of a three-chamber design. The bottom chamber is filled with water, and the coffee powder is placed in the middle chamber. By heating, the hot water and steam pass through the coffee bed, extracting soluble and emulsifiable substances. The air-vapor pressure generated in the bottom chamber drives the extraction process. The beverage will then be collected in the upper section [3,15,25,26]. Another example of a technique that has experienced a recent surge in popularity is cold brew coffee. The cold brew consists of preparing coffee with cold water, usually at room temperature or lower, over longer than other coffee preparation methods. In fact, the steeping time ranges from 8 to 24 h. Therefore, the main differences with the other techniques are the extraction temperature and brewing time. The temperature often significantly influences the aqueous solubility of compounds; hence, brewing temperatures significantly modify the composition of hot and cold brews. In addition, the longer brewing times of cold brew coffee may affect the content of numerous substances [27,28]. The most popular method that exploits the pressured and hot water for preparing a pleasant and short beverage is the espresso coffee.

### 1.3. Espresso Coffee 

The espresso coffee is one of the most widely consumed beverages in the world and mainly in southern Europe and Central America [26]. The Italian word *espresso* suggests that this beverage must be quickly prepared on customer demands or rather extemporaneously prepared after the consumer orders. Another important characteristic of espresso coffee is the use of pressure. The beverage must be prepared not only with hot water, as the main coffee brewing, but also under pressure. Therefore, for the espresso definition, three essential features are required: extemporaneous preparation, fast brewing, and the use of pressure. A typical definition of the espresso is the following: a concentrated polyphasic beverage with a characteristic foam layer (crema) on the surface, prepared by forcing hot water (90 ± 5 °C) under pressure (9 ± 2 bar) into a tamped R&G coffee (called coffee cake) for a short period of time (30 ± 5 s). The cup volume can change from 15 to 50 mL on consumer preferences [5]. The preparation conditions, such as the amount of R&G coffee to brew, the design of the filter basket, the time of extraction, the volume in the cup, etc., change from country to country. For preparing a Certified Italian Espresso Coffee (EC), the preparation and the drink must respect rigid production specifications. The Italian Espresso National Institute emitted these specifications, which were approved by a Third-Party Body, and these are protected and divulged through a product certification (certificate of product conformity Csqa n. 214: 24 September 1999, DTP 008 Ed.1). Some important parameters to follow for obtaining a Certified Italian Espresso Coffee (EC) are: R&G coffee (7 ± 0.5 g), the exit temperature of water from the unit at 90 ± 2 °C, the temperature of the drink in the cup at 67 ± 3 °C, the entry water pressure 9 ± 1 bar, percolation time 25 ± 2.5 s, viscosity at 45 °C > 1.5 mPa, total fat >2 mg/mL, caffeine <100 mg/cup, and the volume in the cup (including foam) at 25 ± 2.5 mL [26,29]. For the EC preparation, numerous machines are commercially available, with different designs and technologies; almost all are composed of three essential parts: the pump, the extraction chamber, and the heat exchanger. The extraction occurs by bringing water to set up the pressure and then compelling it through a heat exchanger where water is led to the desired temperature. Then, hot water proceeds to the extraction chamber, which is composed of heated and fixed parts into which the filter holder is fitted snugly. The filter holder contains a filter basket where the coffee cake is settled. In the extraction chamber, water crosses the perforated disc and is sprayed through the shower evenly over the coffee cake surface. The pre-infusion takes place in the first seconds, where the coffee absorbs some milliliters of water and swells. This allows the coffee surface to reach the required permeability [26], and then the extraction phase begins. The extraction phase is a complex mechanism where several phenomena occur, such as the dissolution of aqueous soluble compounds, forced extraction of some less soluble compounds and physically entrapped molecules (e.g., arabinogalactans), degradation reactions due to heating that can affect the solubility of many substances (e.g., galactomannans), the migration of fine particles, and coffee oil through the water flow, etc. [30]. Compounds with different chemical–physical properties are extracted by this preparation technique, resulting in a complex flavor beverage. In fact, EC contains several classes of compounds, and the main well-known and studied bioactive molecules are alkaloids, phenolic acids, derivatives of phenolic acids, diterpenes, and melanoidins [31]. Many variables related to EC preparation, such as the amount of R&G coffee, particle size distribution (PSD), filter basket, perforated disc, water quality, temperature and pressure of water, percolation time, cake porosity, etc., can play an important role in coffee extraction [5]. For example, Khamitova et al. [8] reported that brewing espresso with a smaller particle size of R&G coffee increased the content of the bioactive compounds, while Salamanca et al. [32] demonstrated that using gradient temperature during espresso preparation generates differences in physicochemical properties of EC. 

Several studies were conducted to obtain deep insight into coffee flavor and to establish which are the most important flavor-active compounds and how preparation and extraction parameters can affect coffee sensorial properties. Researchers have tried to review papers and scientific results on coffee aroma, but to the best of our knowledge, this is the first review focusing on EC aroma profile and key odorants of espresso coffee. Thus, the objectives of the present review were to summarize the research on the characterization of espresso coffee aroma and key-aroma compounds and the influence of preparation variables on EC flavor. In addition, a compendium of the analytical techniques for the determination of major volatile organic compounds (VOCs) in EC was reported. We believe that this review can help to deeply understand the EC aroma profile and how different variables can affect beverage flavor to guide baristas and consumers to obtain the desired EC. Moreover, adjusting the extraction conditions might generate a more eco-friendly consumption of this drink by decreasing the amount of R&G coffee used, and at the same time, maintaining the same product quality. 

## 2. An Overview of EC Aroma: Arabica and Robusta

Coffee aroma is a decisive feature for product quality and consumer acceptance [33]. Aroma volatiles produced during the roasting of coffee beans are the most important quality attribute of coffee, and therefore, they were intensively studied over the past 50 years. The geographical origins of the coffee, different cultivars, processing techniques used, and styles have a great effect on the aroma volatiles of coffee [25]. It is well known that the perceived EC aroma is not only related to the total amount of aromatic compounds extracted during the preparation but also related to other EC machine parameters such as the extraction temperature and the presence/absence of foam [34]. 

### 2.1. Aroma Profile of Espresso Coffee (EC)

Volatile components or volatile organic compounds (VOCs) of coffee have received great attention. More than 1000 volatiles were identified in coffee [25], but only a few of them are considered important to the flavor and aroma characteristics of coffee. These are believed to impact aroma compounds [35,36]. They are classified by chemical classes into pyrazines, furans, aldehydes, ketones, and pyrroles. These compounds are present together with phenols, hydrocarbons, acids and anhydrides, esters, alcohols, sulfur compounds, and others [37,38]. Quantitatively, the main two chemical classes in coffee are furans and pyrazines, while qualitatively, sulfur-containing compounds together with pyrazines are considered the most significant to coffee flavor [25,39]. It was suggested that only from 20 to 30 volatile compounds could be important to the aroma of different kinds of coffee [40,41,42,43,44,45]. These compounds exhibit variable changes in concentration and sensory potency, which explains why different coffee types may have such unique and specific flavors [46]. It was reported that espresso coffee (EC) contains volatile components belonging to different chemical classes such as furans, pyrazines, aldehydes, ketones, phenolic, and sulfur compounds [37,47,48,49].

Among the most abundant chemical classes of volatile compounds in coffee, furans occur [45]. Furans and pyrans, the Maillard-reaction products, are produced from various chemical reactions such as the thermal oxidation of lipids, thermal degradation of *D*-glucose and sugar polymers, thiamine, and nucleotides degradation [50]. Additionally, furanic compounds are considered the major chemical class detected in EC [47,48]. It was reported [47] that EC is richer in furans in comparison with Moka and Neapolitan coffees while they showed high similarity to American coffee. The main differences were found in the following compounds; 2,5-dimethylfuran, furfuryl methyl ether, and 2-furanmethanol acetate. The 2-Furanmethanol acetate was found in higher amounts in EC, while the other two compounds were detected in higher levels in American brew. On the other side, there were no significant differences among the analyzed coffee brews for 2-furnanmethanol and furfurylmethyl sulfide. Among diverse classes of volatile compounds in EC, furans occurred at the highest concentration, followed by phenolic compounds, pyrazines, aldehydes, and ketones [47]. Hovell et al. showed that some furanic compounds could be used to discriminate arabica and robusta coffees, namely 2-furfural, furfuryl alcohol, 5-methylfurfural, and furfuryl acetate [51]. Petisca et al. reported that the furanic compounds abundance in EC from 100% arabica variety was furfuryl acetate followed by furfuryl alcohol, 2-furfural, 5-methylfurfural, and furan [52]. This result was close to that obtained for 100% arabica dark roasted, 100% arabica light roasted espresso samples [48]. Moreover, it was possible to observe that for espresso samples prepared from different arabica and robusta blends with diverse roasting degrees, such as blend roasted, blend darkly roasted, arabica light roasted, and arabica dark roasted, the major furanic compounds were 2-furfural, followed by furfuryl alcohol, 5-methylfurfural, furfuryl acetate, and furan [52]. When aroma was added to the samples, furfuryl alcohol was the major furanic compound, followed by 2-furfural, 5-methylfurfural, furfuryl acetate, and furan. These results concerning the levels of furan were in accordance with other authors for EC [53,54,55,56], although it was reported that furan levels could change from sample to sample, due to many factors, for example, type of extraction method, brewing temperature, and the type of fiber used during sample preparation and roasted coffee beans. Lolli et al. studied the aroma profile of 65 capsules brewed EC belonging to five commercial brands sold in Italy, and eight furanic compounds were found in all samples, except for vinyl furan, which was present at the lowest concentration between these compounds [37]. In 1995, the International Agency for Research on Cancer (IARC) classified furan as type 2B—“possibly carcinogenic to humans” due to their methyl furan metabolites which are considered to be toxic. Recently, in 2016, the IARC reassessed the carcinogenicity of coffee and reclassified it as type 3—“not classifiable as to its carcinogenicity to humans” because the evidence is not sufficient [57]. Furan and its methyl derivatives concentration in the EC beverage depend on many factors such as processing steps, coffee composition, and brewing methods. It was previously reported [57] that capsules brewed coffees exhibited the minimum levels of furan and its derivatives in comparison with other preparation techniques, except for instant coffee, which showed fewer furans levels [58]. Other authors reported opposite data because they detected higher levels of furan and furan derivatives in commercially packed coffee capsules compared to coffee samples from other brewing methods [56]. In the work of Lolli et al. [37], furan was not found in EC samples, in accordance with previous data [57], and concentrations of 2,5-dimethylfuran and 2-methylfuran were found to be in the range of 9.0–112.0 and 45.0–531.0 µg/L for all samples, respectively. Generally, the concentration of methyl furans shows a high variability and, in some cases, results were higher than those reported in the literature [57], however, they were in accordance with others [56,58]. A significant variability among levels of methyl furan derivatives was noted between different brands of EC samples. For example, the samples mixed with flavors such as chocolate and caramel, were more rich in methylfurans, in particular 2,5-dimethylfuran, which exceeded the reported ranges mentioned by some authos [57]. This can be explained by the presence of significant amounts of furans in these flavor additives [58] which consequently increase their levels in the EC brew. On the contrary, ECs from some brands showed significant lower amounts of methyl furans than the others. In conclusion, these results could help to evaluate and establish the furan contents in EC. 

Several phenolic compounds were detected in ECs from different Italian brands. For example, guaiacol was detected in all the samples, but there were no significant differences between the different brands, indicating that concentration patterns of the volatiles belonging to phenolic compounds had scarce significance for the discrimination of aroma differences [37]. Some phenolic compounds, especially guaiacol, 4-ethylguaiacol, and 4-vinylguaiacol [25], produced during the roasting process, are considered very important for coffee flavor [50,59]. In roasted and ground coffee (arabica), phenolic compounds occur from 3 to 56 mg/kg [60], depending on geographical source and variety. These phenolic compounds derive from the thermal degradation of chlorogenic acids, and their contents in roasted and ground coffee are related to the levels of chlorogenic acids in the original green beans. Normally, robusta green coffee contains a higher concentration of chlorogenic acids; hence, the volatiles could be useful in the aroma discrimination of the two coffee species [61,62]. It was reported that, for phenolic acids, guaiacol content represents the main differences between different preparation methods. In fact, its content was lower in EC than in Moka coffee brew [47]. Guaiacol arises from ferulic acid thermal degradation, and its concentration increases with roasting. Therefore, guaiacol yield increases when higher extraction temperatures are used [50].

Pyrazines are a well-known group of molecules that derive as a product of roasting diverse foods, including coffee. This class of molecules is abundant in coffee [25], and they are formed by reactions between carbohydrates and α-amino acids. Alkylpyrazines are considered key aroma components of coffee brew [50]. For example, 2-ethyl-3,5-dimethylpyrazine, 2-methyl-3-*trans*-propenylpyrazine, and 2-ethyl-6-methylpyrazine were detected at low levels in EC [47]. Andueza et al. reported that water extraction temperature of ≥ 96 °C resulted in a higher pyrazine amount during the preparation of EC from arabica. Therefore, higher amounts of some pyrazines in EC are related to the higher water temperature used for the extraction [49]. Lolli et al. [37] detected 12 pyrazines in capsules-brewed ECs from various brands sold in Italy. Among them, 2-ethyl-6-methylpyrazine, 2-ethyl-3,5-dimethylpyrazine, and 2-ethylpyrazine were previously reported as potent key aroma components [63]. Moreover, the levels of pyrazines were statistically different among different brands. Together with thiazoles, pyrazines contain the lowest odor threshold, so they have an important and significant contribution to the coffee aroma. In addition, structurally similar alkylpyrazines from coffee may show similar properties and contribute to the various physiological actions of coffee [64]. A previous study [65] mentioned that high amounts of pyrazines are related to species and cultivars, mainly roasted powder, such as 2,3-diethyl-5-methylpyrazine, 2,3,5-trimethylpyrazine, 3-ethyl-2,5-dimethylpyrazine, and 2-ethyl-5-methylpyrazine, together with other phenolic compounds, pyridine, alkyl- and furfuryl-pyrroles, and *N*-methyl-2-pyrrole-carboxaldheyde. In this study, the levels of alkylpyrazines were higher than those reported for brewed green coffees in a previous work [66]. On the other hand, these concentrations are lower than those reported for roasted brew coffee for arabica and robusta [64], which makes a classification based on cultivars difficult. Moreover, 2,6-diethyl-pyrazine was reported to show the lowest concentration in decaffeinated EC [37], and this result agrees with data reported about decaffeinated ECs. This type of coffee usually possesses lower content of pyrazines, likely due to the decaffeination process [64]. In a previous study, pyrazines were detected at higher amounts in torrefacto roasted EC; this was due to the sugar addition, which enhanced Maillard reactions [67].

Aldehydes arise from the oxidative degradation of amino acids during their interactions with sugars at high temperatures or polyphenols in the presence of polyphenol oxidase. Some aldehydes, such as hexanal, are considered autoxidation products of unsaturated fatty acids by the breakdown of hydroperoxide intermediates [50]. Therefore, taking into account the content of lipids and proteins in green coffee beans, their contribution to the aroma of the coffee brew is not surprising. Lolli et al. reported that the concentration values of 2-methylpropanal, 2-methylbutanal, and 3-methylbutanal aldehydes in capsule-brewed ECs were in good agreement with the previous literature data [66], and there are differences among the type/taste of capsules. However, there was no consistent trend in the content of aldehydes, which could suggest a potential variability in the quality and the perceived aroma from capsule-brewed ECs, especially from different brands [37]. Several aldehydes were reported in EC, such as acetaldehyde, propanal, 2-methylpropanal, 2-methylbutanal, and 3-methylbutanal [47,49,67,68].

Ketones were reported in the EC of arabica and robusta coffee beans [37,47,49]. Many studies reported the presence of 2,3-pentanedione and 2,3-butanedione in EC samples prepared from arabica and robusta natural blend coffees, while other ketones such as *β*-damascenone, 2-butanone, and 2,3-hexanedione, etc., were detected in arabica EC; the relative percentages of these ketones in arabica EC were significantly higher with respect to natural robusta blend EC [10,47,49,67,68,69,70]. The foremost volatile molecule generated from the thermal degradation of furaneol is 2,3-pentanedione, while *β*-damascenone is formed by carotenoids. It was previously reported that ketones were detected in higher amounts in filtered coffee than EC [47,71].

Sulfur-containing compounds, e.g., methanethiol, a mercaptan that derives from methionine pyrolysis, are very important contributors to coffee flavor due to their sensory potency [50]. Methanethiol was found at higher concentrations in espresso with respect to other brewing techniques, probably due to the higher water pressure and the rapid brewing time [47]. In a previous study, Lolli et al. [37] mentioned that 2-(methylsulfanylmethyl)furan and dimethylsulfide were found in the major of the tested EC capsule brands, and this is consistent with the literature [65]. The high variability in the concentration of these sulfur compounds may be caused to the diverse way of processing, the species, packaging, and storage conditions. It is reported that robusta possesses more 2-(methylsulfanylmethyl)furan than arabica [65], but the levels of this compound in ECs significantly varied from capsules of different brands (brand B and D). However, the diverse levels of this volatile agreed with those found in arabica and coffee blends in previous investigations [72], but not being able to discriminate between species, quality differences, and chemical aroma profile.

Several pyrroles such as 1-methyl-1*H*-pyrrole, 1-ethyl-1*H*-pyrrole were reported in EC prepared from arabica coffee [47,69,70]. They are considered as furan degradation products and amino acid derivatives, but they are generated by thermal degradation of Amadori intermediates as well [50]. 1-Methyl-(1*H*-pyrrol-2-yl)-1-ethanone is the major pyrrole, and it seems not to be influenced by the preparation method [47].

Parenti et al. [34] compared different preparation techniques (Bar Machine (BM), Hyper Espresso method (HIP), and I-Espresso System (IT)) in terms of the aromatic profile of the resulting ECs. The main differences in aromatic profile were found in EC produced by the BM system, which is characterized by higher levels of all monitored eight marker compounds (3-methylbutanal, 2-ethyilpyrazine, 2-methylbutanal 2-ethyl-6-methylpyrazinem, 2-ethyl-3,5-dimethyilpyrazine guaiacol, 2,3-pentanedione, and diacetyl), indicating a more characteristic profile than the other preparation techniques. Thus, obtained results proved that the EC produced by the BM system possessed a more intense flavor than the capsule systems. Furthermore, Rocha et al. [73] compared the aroma profile of coffee plunger coffees and EC obtained from diverse blends such as arabica, natural robusta blend, and robusta torrefacto. They identified 37 compounds as follows: 11 furans, 10 pyrazines, 4 aldehydes, 3 phenolic compounds, 2 ketones, 2 pyridines, 2 indoles, 1 ester, 1 lactone, and 1 benzothiazine. The volatile profile was more related to the type of coffee (arabica or robusta) than to the method of preparation (coffee plunger or EC). EC volatiles showed higher relative percentage areas than those of coffee plunger volatiles. From an extensive review of the espresso coffee (EC) literature, a summary of volatiles thought to be of greatest importance to the aroma of arabica and robusta EC is provided in Table 1 along with their relative percentages of area and the literature references. 

### 2.2. Espresso Coffee (EC) Key Odorant Compounds

Although many volatile compounds were reported in coffee, only a few are considered to be key odorants that are responsible for the coffee aroma [40,73,74]. These volatile compounds are reported to be formed during coffee bean roasting [47]. There were numerous studies focused on the factors that influence these key odor compounds that actually contribute to the aroma of EC. Among these, the influence of the botanical varieties (robusta and arabica), roasting types, grinding [67], milk addition [69], and commercial brands [37] on EC flavor. Moreover, the impact of temperature and pressure on the chemical and sensorial characteristics of EC was studied [40]. Many studies used (GC-MS), to analyze the aroma of EC [37,40,48,69,75,76]. Although GC-MS is considered a useful analytical tool, it does not give any odor descriptions of volatile compounds, and therefore, it does not distinguish and identify the compounds which are responsible for the aroma. For this, gas chromatography-olfactometry (GC-O) could be considered a valuable system to determine the main key odorants. GC-O refers to the techniques that combine the use of the human nose as a detector with the usual gas chromatographic separation [77]. The data that are generated by GC-O analysis can then be evaluated using CharmAnalysis^TM^ (combined hedonic aroma response measurement), aroma extract dilution analysis (AEDA), surface nasal impact frequency (SNIF), and Osme (from the Greek word meaning odor). Several studies have used GC-O to identify the key odorants of EC aroma. For example, Michishita et al. [76] evaluated the retronasal aroma of espresso by correlating the sensory evaluation data with data obtained from gas chromatography-olfactometry (GC-O, CharmAnalysis^TM^). In this work, the volatile compounds of various types of espressos from six production countries, with three roasting degrees, were collected with a retronasal aroma simulator (RAS) and examined by GC-O and E-nose. The charm values of 10 odor descriptions obtained from GC-O analysis exhibited significant (*P <* 0.05) differences between both roasting degrees and origins. In this study, 36 potent odorants were detected by GC-O analyses. Significant differences in the origin were found in all odor descriptions in the three roasting ranges, except for green–blackcurrant among the light-roasted sample. A previous study of Akiyama et al.[69] was based on the calculation of odor spectrum values (OSV) using GC-O methods and application of other techniques such as Charm analysis. The study reported that when milk was added to espresso, aroma release was generally suppressed. Maeztu et al. [75] studied the aromas of three EC samples from different botanical origins and roasting types, and reported 13 key odorants, which were quantified and correlated with their flavor notes: some aldehydes such as acetaldehyde and propanal with a fruity aroma, diones with buttery odor, sulfur compounds such as methanethiol with a freshness aroma, and pyrazines with earthy/musty, roasty/burnt, and woody/papery flavors. Therefore, a summary of key odorants reported to be the most important to the flavor of EC is provided in Table 2. These are listed according to their chemical classes together with their odor description.

Many aldehydes were identified and quantified in EC as key odorants. Acetaldehyde and propanal were highly significantly correlated with fruity [75] and green odor [50] in the coffee brew. Moreover, a marked correlation was found between the fermented aroma and 2-methylbutanal [75]. 2-Methylpropanal, 2-methylbutanal, and 3-methylbutanal, which are the Strecker degradation products of the branched-chain amino acids (BCAA), were the key odorants responsible for malty and fermented flavors in ECs [37,40]. Strecker degradation products were perceived more often in fine and very fine arabica/robusta 20:80, natural roasted ECs compared to the other EC samples [67]. Another compound, hexanal, was also described as the main component responsible for the green note [50]. Generally, aldehydes showed the highest content in espresso brew with respect to Neapolitan and Moka coffee brews [47].

The ketones were reported to generate buttery and creamy attributes in ECs [37,40,75]. Among different identified ketones, 2,3-butanedione and 2,3-pentanedione were evaluated as key odorants; these compounds were correlated with the buttery flavor in EC [75], and *β*-damascenone is responsible for the fruity odors [47]. Higher concentrations of ketones were detected in filtered coffee brew rather than espresso [47,71,74].

Although sulfur-containing compounds, including thiols, occur at relatively low concentrations, they are considered one of the major factors for coffee flavor [25]. Among these, methanethiol has an intense cabbage-like, cheese, garlic-like, and sulfur odor [47]. Although it has been described as an unpleasant odor, methanethiol is generally related to the freshness flavor in EC due to its rapid degradation during coffee brew oxidation [67,75,78]. It was observed that methanethiol produced a synergistic effect with acetaldehyde, increasing the fruity flavor, which might also explain the correlation found between methanethiol and the fruity odor [75,81]. An important compound, 2-furfurylthiol, was reported to exhibit a strong roasted aroma [79]. This compound was also considered by many researchers as a key odorant compound in coffee [42,76,80,82] and was reported previously in EC [76]. Other thiols, such as 2-methyl-3-furanthiol and 3-methyl-2-butene-1-thiol, were also identified in coffee beverages, and they possess very low sensory thresholds while exhibiting meaty notes. Moreover, 3-methylthiophene [59] and 2,4-dimethyl-5-ethylthiazole occur in coffee at significant sensorily levels, and they are described as having roasted and meaty flavors [25].

Pyrazines and furans are the major compounds in coffee and also are the main classes responsible for the coffee characteristic aroma [63]. Pyrazines were previously correlated with the roasty and earthy/musty aromas of roasted coffee and coffee brew [41,83]. Among them, the alkylpyrazines are considered the main key odorants [50]. Ethylpyrazines and ethenylalkylpyrazines were also reported to contribute to the earthy aroma of robusta [25]. The compound 3-isobutyl-2-methoxypyrazine was found at low levels in roasted and ground coffee (arabica), but it was reported to possess a significant impact on the aroma [42]. 2-Ethyl-3,5-dimethylpyrazine and 2,3-diethyl-5-methylpyrazine are also considered two important aroma compounds which contribute to coffee flavor [44,67,84]. Maeztu et al. [75] reported that among 13 identified pyrazines in ECs, three were quantified as key odorants such as 2-ethyl-3,5-dimethylpyrazine, 2-ethylpyrazine, and 2-ethyl-6-methylpyrazine. These compounds are releated to the woody/papery, roasty/burnt, and earthy/musty flavors, respectively. From those, 2-ethyl-3,5-dimethylpyrazine is considered to be one of the most powerful odorants in coffee, which might be responsible for the woody/papery and roasty/burnt flavor [41]. However, earthy/musty aroma seems to be more affected by 2-ethyl-6-methylpyrazine in EC samples.

Furans are found in sensorily or aroma significant active concentrations in roasted coffee [60,80,85,86]. Volatile furans are characterized by malty and sweet roasted aromas [80,87]. They possess relatively high sensory thresholds compared to other classes of coffee volatiles [87], but they are still considered of importance to coffee flavor since they occur at high concentrations. The concentration of furan and its methyl derivatives in EC are influenced by many factors, such as coffee composition, processing steps, brewing methods, and the presence of flavor additives or aromatic substances [37]. These molecules with pyrans are reported to be the main responsible components for the known aroma of coffee brew [50]. The smoke–roast odor given by 2-((methylthio)methyl)furan and 2-furfurylthiol (2-furanmethanethiol) was predicted as one of the main odors affecting the sensory characteristics of the overall RAS aroma in EC [76]. Moreover, Furanones are among the major flavor contributors in EC, such as 4-hydroxy-2,5-dimethyl-3(2H)-furanone and 4,5-dimethyl-3-hydroxy-2(5H)-furanone, which are thought to be responsible for the sweet caramel aroma of EC [76].

The phenolic compound guaiacol was reported to be responsible for phenolic and spicy aromas [41] and also phenolic and burnt flavors [88]. In a previous study, a highly significant correlation was found only between this compound and the spicy flavor in EC samples [75]. In fact, the increase in guaiacol seems to be higher when exposed to higher extraction temperatures [50,89].

### 2.3. Sensory Attributes of Espresso Coffee and Their Relation to the Volatile Profile

Volatile profile only is not enough to explain the importance of key compounds and, importantly, the nature of their contribution to coffee flavor. To deeply understand the relation between sensory properties and chemical composition of EC, researchers may use chemical analysis in conjunction with certain chemometric tools (i.e., multivariant statistical methods) for assessing flavor quality. Commonly used techniques are a principal component analysis (PCA) and a partial least squares (PLS) regression [25,90]. Some studies on EC flavor have focused on the determination of the relatively important volatiles to the EC flavor to identify those molecules that have a real olfactive impact on EC aroma. A correlation was established between key odorants and flavors in different ECs by the application of multivariate statistical methods, and the sensory flavor profile was performed using a selected and trained panel of judges [75].

Principal component analysis (PCA) was used to examine differences between several coffee brews (Neapolitan, espresso, American, and Moka coffee brews). The diverse preparations were clearly separated by PCA, indicating that each coffee drink shows some chemical characteristics. Acetaldehyde, metanethiol, 2-furanmethanol acetate, butanal, and 2-methylpropanal characterized EC [47]. Another study [10] mentioned that the ECs prepared at three pressures were perfectly separated by PCA. Coffees prepared at 9 atm showed a high percentage of key odorants related to freshness and fruity, malty, and buttery flavors. Moreover, Parenti et al. used a multivariate analysis to show that different extraction techniques produce different ECs [34]. Rocha et al. [73] used PCA to investigate the main differences between the diverse coffees and to establish a correlation between the botanical origins, blending and brew methods, and volatile components. The study found 2-methylfuran as the typical component of EC. In addition, the arabica brews were characterized by furfuryl acetate, while the robusta brews were characterized by 2-methylbutanal, 2-ethyl-5-methylpyrazine, trimethylpyrazine, and 3-ethyl-2,5-dimethylpyrazine [73]. Maeztu et al. [75] reported that a correlation was achieved between key odorants and flavors in various ECs by the application of multivariate statistical methods and sensory flavor profiles. Furthermore, Lolli et al.[37] studied the volatile profile of EC capsules, and findings were submitted to PCA. They did not find significant variability between EC samples of the same brand except for those modified capsules with the addition of specific flavor additives. This study also used a partial least squares discriminant analysis (PLS-DA) to show that capsules from one brand possessed the highest pyrazine concentration, and therefore was characterized by a typical aroma and a stronger note than those from the other brands.

## 3. EC Machine Parameters and Their Influence on the EC Flavor

**Figure 1:**
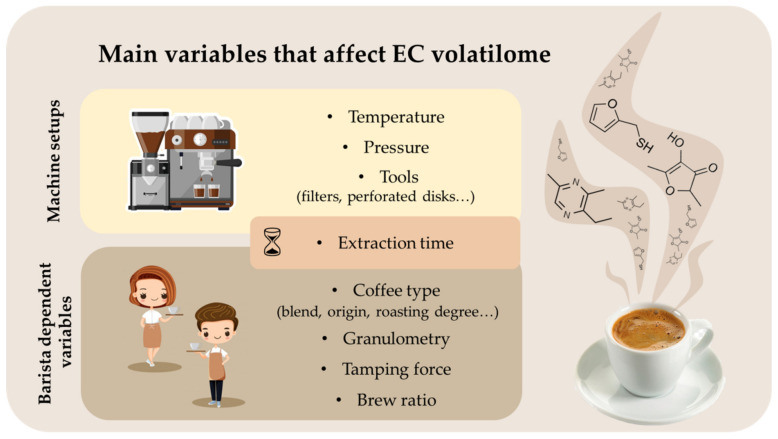
Main preparation variables that influence the EC volatilome.

EC flavor is the result of a balance in the regulation of many variables during the extraction process. These variables are interlinked with each other, and the adjusting of one of them results in modifying all others. Therefore, the preparation of the best EC in terms of sensory profile and customer acceptance is a hard challenge. In this chapter, some of the most studied variables are taken into consideration to give an overview of which parameters should be controlled to prepare the desired EC. In detail, extraction time, water temperature and pressure, and granulometry of coffee powder (particle sizes) were considered. Figure 1 reports the main preparation variables which can affect the espresso coffee flavor and, therefore, its volatilome. These were divided under machine setups (water temperature and pressure and tools), barista dependent variables (coffee type, granulometry, tamping force, and brew ratio), and mixed one (extraction time).

### 3.1. Extraction Time

The extraction of compounds responsible for the EC flavor profile is dependent on the contact time between water and coffee. In the first seconds of extraction, highly soluble compounds such as sugars, organic acid, caffeine, etc., are extracted for more than 90% of the extraction yield. Moreover, the extraction of compounds responsible for the aromatic profile decreases when the time extraction increases, as well as those of soluble compounds [3,11]. In the same way, more than 70% of antioxidants, except for dicaffeoylquinic acid (diCQA), are extracted in 8 s in espresso coffee. This data is in contrast with antioxidant extraction in filter coffee, which takes longer (75 s) but with higher antioxidant content, especially for less polar compounds such as diCQA [91]. A little more time (10 s) is needed for the extraction of 95% of volatile organic compounds (VOCs) with a concentration peak between 2 and 24 s [92]. Finally, less soluble compounds, such as bitter and astringent compounds, are extracted using a longer extraction time or a higher water content [93].

### 3.2. Water Temperature

Temperature is a key parameter in the extraction process because of its double effect. In fact, on one side, it is responsible for the increased mobility of water molecules, with subsequent increase in extraction from coffee [93], and on the other side, it could be responsible for the release of VOCs, indispensable for the sensory profile. A balance between the increment of molecule extraction and the possible loss of VOCs is required when choosing temperature to maintain the coffee sensory profile. Moreover, a higher water temperature results in higher total solids and caffeine content. The foam index also increases with the temperature [94]. Moreover, using an increasing temperature ramp (88–93 °C), there is an increment in caffeine, acidic compounds, and chlorogenic acid content, which gives to EC astringency, bitterness, good crema color, well-balanced aroma intensity, body, and flavor. The use of a decreasing temperature ramp (93–88 °C) results in decreased 5-dicaffeoylquinic acid (5-CQA) content, decrement in foam index, viscosity, body, and level of pleasant odors, and the increment in bitterness and astringency [32]. For what concerns VOCs, using higher temperature results in a higher release of these compounds [95]. In particular, the use of water at a temperature of ≥96 °C makes substances appear, such as guaiacol and pyrazines [49]. Caprioli et al. studied the effects of temperature on EC sensory profile using two types of coffee machines, set with different conditions and demonstrated that a good sensory profile is maintained in EC prepared in 10 s with a water temperature of 101 °C, which increases slowly, and a pressure of 8 atm with respect to an EC prepared in 25 s with a water temperature of 92 °C and a pressure of 9 atm [40].

### 3.3. Pressure

Pressure is a very important parameter in EC preparation because it allows water to penetrate the packed coffee for the extraction process [96]. A higher pressure, in fact, is related to a higher content in aromatic compounds that evaporate less when pressure is applied. The application of 11 bar of pressure increases the VOCs content in EC, with peaks of concentration during the first 10 s [95]. The extraction of some biomolecules also increases with the pressure. In fact, trigonelline, caffeine, and nicotinic acid are most extracted using 7−9 bar pressure at 92 °C [97], and the same for total lipids, which are more extracted with pressures that reach 12 bars [34]. Pressure is also responsible for foam and crema formation. In fact, the carbon dioxide in coffee is forced into the water by the pressure and, when released, it forms the typical crema above the coffee [93]. In particular, ECs prepared at 9 bar pressure have a high foam consistency, with high aroma intensity [10,40]. The increment of the pressure until 11 bar leads to an increment of the bitterness, astringency, and aftertaste intensity, making these ECs less palatable [10]. However, the use of pressure too high (20 bar) results in decreased caffeine and chlorogenic acid content [98].

### 3.4. Particle Sizes

The influence of the particle size in the extraction process is related to the availability of the surface area obtained after the grinding process. This is an aspect not to be underestimated. In fact, the use of coarse particles results in reducing the extraction yield because of the too high flow of water between the particles of coffee [99]; on the other hand, instead, the use of small particles can clog the filter basket and can lead to an over-extraction, due to a longer contact between water and coffee particles [100]. Severini et al., 2015 demonstrated that the use of finer particles leads to an increment of the extraction of compounds such as caffeine or compounds responsible for the aromatic profile of coffee [11]. The particle sizes are not constant in coffee samples because it depends on the moisture content and the roasting degree during the grinding process. In particular, the higher is the roasting degree, the higher is the porosity and the brittleness of particles [5]. For what concerns the moisture content instead, the higher the water content is, the bigger the particles are [100]. For these reasons, it is not easy to determine the particle size changes during the grinding process in order to determine its influence on sensory profile in the EC. However, some studies take into consideration the pores between the particles (intergranular) and into the particles (intragranular) in order to assess their influence on the extraction process [96,101,102,103]. Table 3 summarizes the influence of main parameters on EC extraction and flavor.

## 4. Analytical Methods for the Determination of VOC in Espresso Coffee

In the scientific literature, several articles on the aroma of roasted and ground coffee and coffee beverages are reported. However, solely a few of them focused specifically on EC aroma. EC aroma can be mainly due to the surface foam, which can trap volatilized aromas and dose their release in the atmosphere [5]. Sensory descriptive methods performed with a panel of judges can describe the real EC aroma profile. In addition, different analytical techniques were used to study volatiles fingerprints of the captivating aromas of EC. The main analytical methods used for VOCs analysis in EC are reported in Table 4. Most of them can be classified under three major analytical techniques: gas chromatography (GC), proton-transfer-reaction mass spectrometry (PTR-MS), and electronic nose sensing (EN).

### 4.1. Gas Chromatography Methods for the Analysis of VOC in EC

GC techniques are highly used for the analysis of volatile compounds in EC and involve a sample preparation step using different VOC extraction systems. Extracted VOC are then separated through a GC column and detected by specific detector systems [104].

#### 4.1.1. Sample Preparation Techniques

**Figure 2:**
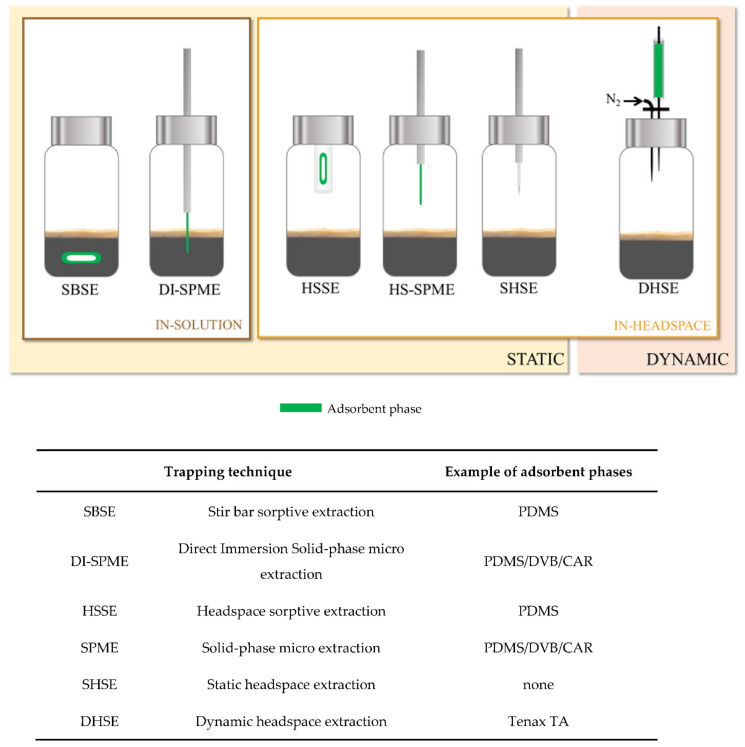
Schematic representation of the six volatile trapping techniques used for VOCs extraction in EC. The adsorbent coatings are highlighted in green. Abbreviations are explained in the table above.

Various sample preparation techniques were used for the extraction of aroma-active compounds in EC (Figure 2).

##### Solvent Extraction Method

The preparation of EC samples for GC analysis can be performed by a solvent extraction method followed by direct injection into the GC injection port. In this case, coffee brew samples are extracted with an organic solvent (dichloromethane, hexane). The obtained extract can be dehydrated and then concentrated under N_2_ for analysis [110]. This method allows the extraction of a wide spectrum of compounds from volatile to semi-volatile compounds. However, the solvent evaporation step, which is essential in the solvent-assisted extraction technique, results in the loss of some volatile compounds and the formation of new compounds not present in the original sample [111].

##### Static Headspace Extraction

Static Headspace (SHS) extraction is one of the most used sample preparation techniques for analyzing volatile compounds from coffee brews. In SHS, a fixed volume of EC (4−8 mL) is introduced in a vial (10−20 mL), immediately closed and sealed, at a precise temperature (40−70 °C) and a fixed time (30-60 min) [105,112]. At the equilibrium, a volume of coffee headspace (1−3 mL) is collected and injected into the GC column. Unlike other GC preparation techniques, in SHS extraction, all volatile compounds of EC are introduced into the GC system in a non-discriminative way. This is highly suitable since the introduced sample is close to the realistic representation of the EC aroma as perceived by the consumers [111]. Moreover, SHS extraction can easily be performed to extract volatile compounds without using specific sorbents (solid-phase materials), solvents, or reagents [113]. However, SHS presents an issue of sensitivity associated with the low levels of the extracted compounds.

##### HS-Solid Phase Microextraction (HS-SPME) Technique

HS-SPME is a commonly adopted sampling technique for the extraction of VOC in EC. This sampling technique consists of the transfer of VOC from the sample matrix to the HS followed by their extraction by the coating phase of an SPME fiber [114]. Although some variations in the methodology parameters were used, the HS-SPME applied to coffee brew involves the heating of the samples (50−60 °C) for a fixed time (5−30 min) under agitation. Then, a fiber is introduced in the sample HS for 20−30 min of absorption and is thermally desorbed in the GC injection port. Polydimethylsiloxane (PDMS) and Divinylbenzene–Carboxen–Polydimethylsiloxane (DVB/CAR/PDMS) are the most used SPME fibers for EC applications [8,37,66]. HS-SPME is mostly used to assess the volatomic fingerprint of EC in addition to multivariate statistical tools to differentiate coffee brews and thus ascertain the coffee quality and authenticity [115]. Genovese et al. [106] compared the profile of 24 key aroma compounds from typical coffee brews under mouth-simulated conditions through an HS-SPME-GC-MS method. Compared to other HS sampling techniques applied to EC, HS-SPME has a higher sensitivity and selectivity. However, the volatile profile of EC after HS-SPME is highly influenced by the SPME fiber coating composition [116].

##### Dynamic Headspace (DHS) Technique

The use of DHS was reported for the extraction of volatile compounds from EC samples through a Purge-and-Trap (PT) sampling [117]. In PT application, an inert gas, especially nitrogen (N_2_), is purged to the sample at a specific flow rate to extract VOCs, which are collected into an adsorbent trap. The trapped compounds are then desorbed using a thermal desorption unit (TDU). Different from other HS techniques, DHS results in a wider aroma profile, also allowing the extraction of hydrophilic and low vapor pressure volatile compounds even at ultra-trace levels. Ochiai et al. proposed a multi-volatile method (MVM) consisting of three different DHS samplings in the same coffee brew sample, which allows the extraction of 658 volatile compounds [118].

##### Other HS Sampling Techniques

Other less used VOC extraction techniques were applied on EC, including headspace sorptive extraction (HSSE) and stir bar sorptive extraction (SBSE). These techniques are based on the trapping of VOC on a polymer-coated magnetic stir bar placed either in the sample HS (HSSE) or in the liquid sample (SBSE) [119]. Bicchi et al. analyzed the VOC profile of arabica coffee brew and reported that the performance of HSSE and SBSE was higher in terms of recoveries compared to SHS and HS-SPME due to the high quantity of the coating polymer and thus a higher sorptive phase volume [120]. However, the coating phase in these techniques is limited to PDMS and EG-Silicone, offering a reduced spectrum of volatiles compounds extracted with a low recovery for polar compounds [121].

Pacheco-Fernandez [122] reported the use of Direct Immersion SPME (DI-SPME) in coffee brew analysis. This technique follows the same principle of SPME except for the immersion of an adapted fiber coating in the liquid sample. However, their study was not dedicated to VOC analysis but to the extraction of polycyclic aromatic hydrocarbons (PAHs) in coffee brew samples.

#### 4.1.2. Gas Chromatography (GC) Separation

After extraction, VOCs are introduced into a GC column and separated according to their polarity and vapor pressure differences. Various GC columns were reported for the analysis of EC volatiles. The most used include polar columns made of polyethylene glycols such as DB-WAX, HP-FFAP, SolGel-WAX, SUPELCO WAX, and ZB-WAX columns [8,37,115,123]. However, the use of non-polar columns such as DB-5, HP-1, and HP-5MS columns, were also reported allowing a good separation of coffee brew volatiles [122,124]. The application of comprehensive two-dimensional gas chromatography (GC x GC) was also reported in a coffee brew analysis [125]. This separation technique, exploiting the different polarities of two combined columns, improves the separation of volatiles, allowing the detection of a higher number of VOC in coffee [126].

#### 4.1.3. GC Detecting Systems in EC Analysis

After separation through a GC column, volatile compounds are revealed by specific detecting systems, which can be used alone or in combination. The main detectors reported in the VOC analysis of EC and other coffee brews include a Mass Spectrometry Detector (MSD), Flame Ionization Detector (FID), and Olfactometry Detector (OD).

GC-MS is the most applied system for the analysis of aroma-active compounds in EC with the use of a single Quadrupole Mass Spectrometer (QMS) as principal mass analyzers [10,11,40,67,105]. The detection of VOC through the QMS involves their ionization through an electron impact (EI) ionization mode (70 eV) with the possibility to detect compounds in the scan mode (40–400 *m/z*) [127] or single ion monitoring mode (SIM) [122]. The QMS offers structural information on the detected compounds allowing to identify the VOC by comparing their retention indexes (RI) and mass spectrum (MS) with reference standards. Moreover, the QMS application allows a comparison of the results obtained in the coffee analysis from different studies since the use of a standardized ionization mode and the disposal of reference libraries with reported compounds mass spectral data (NIST, WILEY, ADAMS, etc.) [128]. Newer studies report the use of more sophisticated mass analyzers such as the Quadrupole-Time-of-Flight Mass Spectrometry analyzer (Q-ToF) [107], which provides a better mass scan efficiency and accuracy to classic qMS. However, the application of these High-Resolution MS was poorly reported in coffee analysis and could represent an analytical innovation for future research on coffee volatomics.

Besides, GC-MS systems can be used in combination with FID for semi-quantitative analysis of coffee aroma-active compounds due to the stability of the latter, giving a more proportional response to the number of organic compounds burnt independently of compound structures. Semi-quantification is calculated by the ratio of each VOC peak area with the peak of the internal standard [107].

GC-effluent sniffing (GC-O) by trained panelists was applied to coffee analysis to determine the volatile compounds playing an important role in coffee brew flavor [129]. Indeed, unlike GC-MS and GC-FID, which allow the identification and quantification of VOC, GC-O offers the advantage of assessing the odor perception and intensity of each eluting VOC [25]. The combination of these three detecting systems can thus provide a wider assessment of EC volatomics [126].

### 4.2. Electronic Nose (EN) Sensing in the VOCs Analysis of EC

In the last decade, EN-related research topics have significantly increased, especially in the food and beverages industry. Known as also the aroma sensor, mechanical nose, multi-sensor array, artificial nose, odor sensing system, or electronic olfactometry, EN technology is inspired by the sense of smell [130]. EN is constituted of three parts: sample delivery, detecting system, and data processing systems [131]. DHS and SPME are the most commonly used GC sampling techniques; they are able to extract VOCs from HS samples and constitute the delivery systems. Extracted VOCs are transferred to the detecting system, which is made of sensors that cause electronic responses reacting with VOCs that are transformed into digital values [130]. Metal-oxide sensors (MOS) are the most utilized sensor types in EN analysis of EC aroma compounds. The EN sensing applications in EC include not only quality studies but also EC extraction parameters optimization and discrimination of EC types. However, EN sensing is mainly used for the characterization of the overall aroma pattern of EC samples, so it is generally coupled with other analytical techniques such as GC, which can qualitatively and quantitatively characterize EC odor-active compounds.

Severini et al. proposed a method based on HS extraction and EN analysis equipped with six MOS sensors to study the changes of the overall aromatic profile of EC for better management of the brew quality. Using EN, it was possible to prove that extraction time and grinding level significantly affect the overall aromatic profiles of EC samples [11]. Michishita et al. performed an analysis using an EN equipped with 18 MOS sensors contained in 3 MOS sensor chambers to monitor the aroma profile from prepackaged chilled espresso beverages, proving that this technique was significantly effective in the evaluation of RAS aromas [76].

### 4.3. Proton Transfer Reaction-MS (PTR-MS) Techniques

PTR-MS is a Direct Injection MS (DI-MS) technique with increasing application in coffee brew volatomic studies. This increase in interest is mostly attributable to its various advantages in VOC analysis, including the absence of sample preparation and the possibility to directly quantify compound levels without the need for a calibration standard [116]. Moreover, coupled with Time-of-Flight (ToF) mass analyzers, PTR-MS provides higher sensitivity and higher mass resolution analysis [132]. Beyond these properties, the use of PTR-ToF-MS can provide interesting results in real-time analysis, allowing to monitor the kinetics of aroma-active compounds in coffee brew during processes such as coffee preparation or coffee drinking [92]. Indeed, time-based flavor generation and intensity are important olfactory characteristics to understand the aroma quality and the in vivo consumer perception of coffee brew. Zanin et al. [108] proposed a PTR-ToF-MS method to assess the release of aroma-active compounds during instant coffee reconstitution. This method allowed to monitor the kinetics of VOCs generated (time-intensity) during reconstitution in order to develop new coffee formulations with enhanced flavor intensity and release quality [109].

## 5. Conclusions

This review focused on the aroma profile of EC and how different preparation conditions could influence it. In addition, the present survey also reported the main analytical technique to analyze EC volatiles. Furans were the most abundant chemical classes reported in EC, while sulfur compounds are considered the main reason for coffee flavor, although they occur at relatively low concentrations. The temperature of extraction, together with particle sizes, were the main variables that affect the EC aroma even if the extraction time seemed significant too. While various analytical instruments were employed to analyze the aroma profile of EC, HS-SPME-GC-MS, together with the GC-O technique, were the most common systems chosen or selected for extensive characterization of volatiles and odor-active compounds in this drink. Considering the importance of the aroma profile as a quality index of EC preparation that also influences consumer acceptance, it is unusual that this is the first review on this field. This highlights the novelty of this survey again. A high number of volatile compounds are produced during coffee processing, especially during roasting, but solely during the extraction process, these volatile molecules are transferred from roasted and ground coffee into the cup. In this phase, it is fundamental to understand which are the main drivers for modulating the migration of aroma from coffee powder into a cup. It is evident that knowing the aroma profile of EC and the main odor-active compounds is essential to study how different preparation variables can be set according to the desired aroma of the resulting cup. The adjusting of the main variables such as temperature and water pressure, particle sizes of coffee powder, machine tools, and brew ratio, which simple as they are, can generate a higher quality beverage and can facilitate baristas and consumers to produce the desired drink. Moreover, the optimization of EC extraction could allow a more eco-friendly EC preparation and consumption using lower coffee powder to produce the same quality of the product.

## Figures and Tables

**Table 1:** Volatile organic compounds (VOCs) identified in different varieties of EC and their relative percentages of the area (% area) according to the literature.

No.	Compound Name and Class	Arabica (% Area)	Blend (Robusta-Arabica (80:20)) (% Area)	References
	**Aldehydes**			
1	Acetaldehyde	0.17–0.49	0.34–0.40	[10,47,49,67,68]
2	2-Methylpropanal	0.65–2.46	2.00-2.83	[10,47,49,67,68]
3	Butanal	0.08–1.16		[47,70]
4	2-Methylbutanal	1.25–2.35	1.26–2.01	[10,47,49,67,68,69,70]
5	3-Methylbutanal	0.59–3.78	2.35–3.38	[10,47,49,68,69,70]
6	Hexanal	0.02–0.08	0.05–0.1	[10,47,49,67,68]
7	Propanal	0.54–0.71	0.47–0.55	[10,49,67,68]
	**Pyrazines**			
8	2-Ethyl-6-methylpyrazine	0.02–1.73	0.04–0.08	[10,47,49,67,68,69,70]
9	2-Ethyl-5-methylpyrazine	1.03–2.9		[47,69,70]
10	2-Propylpyrazine	0.13–0.48		[47,70]
11	2,6-Diethylpyrazine	0.34–0.48		[47,70]
12	2-Ethyl-3,5-dimethylpyrazine	0.01–2.30	0.04–0.08	[10,47,49,67,68]
13	2-Methyl-3-trans-propenylpyrazine	0.38		[47]
14	Pyrazine	0.24–0.44		[69,70]
15	2-Methylpyrazine	2.45–4.08		[69,70]
16	2,5-Dimethylpyrazine	1.43–1.45		[69,70]
17	2,6-Dimethylpyrazine	1.61–1.66		[69,70]
18	2-Ethylpyrazine	0.06–1.61	0.11–0.17	[10,49,67,68,69,70]
19	Trimethylpyrazine	0.67-1.11		[69,70]
20	3-Ethyl-2,5-dimethylpyrazine	1.04–1.38		[69,70]
21	2,3-Dimethylpyrazine	0.28		[70]
22	2-ethyl-3-methyl pyrazine	1.12		[70]
23	5*H*-5-Methyl-6,7- Dihydrocyclopentapyrazine	0.26		[70]
24	Acetyl pyrazine	0.06		[70]
25	1-(6-Methyl-2-pyrazinyl)-1-ethanone	0.18		[70]
	**Furans**			
26	2,5-Dimethylfuran	0.21–0.32		[47,69,70]
27	Furfuryl methyl ether	0.22–1.41		[47,70]
28	Furfurylmethyl sulfide	0.61–7.47		[47,70]
29	2-Furanmethanol acetate	9.46–37.35	0.17	[47,48,70]
30	2-Furanmethanol (furfuryl alcohol)	7.84–18.00	21.00	[47,48,69,70]
31	2-Methylfuran	1.15–2.10		[69,70]
32	3-Methylfuran	0.15		[69]
33	2-Ethenyl-5-methylfuran	0.29		[69]
34	2-(Methoxymethyl)furan	0.64		[69]
35	Dihydro-2-methyl-3(2*H*)-furanone	1.29		[69]
36	2-Furancarboxaldehyde	7.65		[69]
37	2-[(Methylthio)methyl]furan	1.27		[69]
38	Furfuryl formate	0.29–3.59		[69]
39	1-(2-Furanyl)-ethanone	2.33		[69]
40	Furfuryl acetate	15.34		[69]
41	5-Methyl-2-furancarboxaldehyde (5-Methylfurfural)	4.77–7.90	8.20	[48,69,70]
42	2,2’-Methylenebisfuran*c*	0.82		[69]
43	2-(2-Furanylmethyl)-5-methylfuran	0.40		[69]
44	2,3-Dihydro-6-methylthieno[2,3*c*]furan	0.91		[69]
45	Furan	0.21–0.40	0.03	[48,70]
46	2-vinylfuran	0.24		[70]
47	*cis*-2-Methyl-5-npropenylfuran	0.13		[70]
48	Furfural	2.15–10.00	11.00	[48,70]
49	Furaneol	0.15		[70]
50	2-Acetylfuran/2-furfuryl methyl ketone	0.40		[70]
51	1-(2-Furyl)-2-propanone	0.54		[70]
52	Furanmethanol acetate	9.46		[70]
53	2-Furanmethanol propanoate	0.90		[70]
54	2-furfuryl furan	0.76		[70]
55	Dihydro-2(3*H*)-furanone	0.59		[70]
56	1-(2-Furyl)-butan-3-one	0.29		[70]
57	5-Methyl-2-furfurylfuran	0.61		[70]
58	3,4-dimethyl 2,5-furandione	0.09		[70]
59	2,3-dihydro-6-methylthylthieno furan	0.62		[70]
60	2-Acetyl-5-methylfuran	1.48		[70]
61	Difurfuryl ether	2.59		[70]
62	2-Vinyl-5-methylfuran	0.23		[70]
	**Ketones**			
63	2,3-Pentanedione	0.59–2.37	0.42–0.53	[10,47,49,67,68,69,70]
64	*β*-Damascenone	0.06		[47]
65	2-Butanone	0.50–0.86		[69,70]
66	2,3-Butanedione	0.46–1.11	0.32–0.34	[10,49,67,68,69]
67	2,3-Hexanedione	0.69		[69]
68	2,3-Butanedione (diacetyl)	0.32		[70]
69	2,4-Dimethyl-3-pentanone	0.23		[70]
70	3,4-Hexanedione	0.17		[70]
71	3-Hydroxy-2-butanone	0.13		[70]
72	1-Hydroxy-2-propanone	0.50		[70]
73	2-Methyl 2-cyclopenten-1-one	0.09		[70]
74	1-Hydroxy-2-butanone	0.08		[70]
75	3,3-Dimethyl 2-butanone	0.71		[70]
76	1-(Acetyloxy) 2-butanone	0.71		[70]
77	Ethylcyclopentenolone	0.37		[70]
78	3,5-Dimethyl cyclopentenolone	0.12		[70]
79	2-Cyclopenten-1-one, 2-Hydroxy-3-methyl	0.24		[70]
80	3-Ethyl-2-hydroxy-2-cyclopenten-1-one	0.23		[70]
81	3-Hydroxy-2-methyl-4*H*-pyran-4-one	1.35		[70]
	**Alcohols**			
82	2-Methyl-3-Pentanol	0.07		[70]
83	3-Penten-2-ol	0.07		[70]
84	Phenylethyl alcohol	0.21		[70]
	**Acids**			
85	Acetic acid	2.30		[70]
86	Propanoic acid	0.10		[70]
87	*iso*-Valeric acid	0.72		[70]
88	3-methyl-2-butenoic acid	0.12		[70]
	**Esters**			
89	Methyl acetate	0.87		[69]
90	Acetol acetate	2.01		[70]
	**Pyrroles**			
91	1-Methyl-(1*H*-pyrrol-2-yl)-1-ethanone	1.88		[47]
92	1-Methyl-1*H*-pyrrole	0.47–1.73		[69,70]
93	1-Ethyl-1*H*-pyrrole	0.25		[69]
94	1*H*-Pyrrole	0.40–1.51		[69,70]
95	1-Methyl-1*H*-pyrrole-2-carboxaldehyde	1.14		[69]
96	1-(2-Furanylmethyl)-1*H*-pyrrole	1.61		[69]
97	2,5-Dimethyl-1H-pyrrole	2.42		[70]
98	2-Formyl-1-methylpyrrole	1.31		[70]
99	2-Formyl-4,5-dimethylpyrrole	0.39		[70]
100	*N*-furfuryl pyrrole	3.45		[70]
101	Acetyl pyrrole	1.97		[70]
102	1*H*-Pyrrole-2-carboxaldehyde	1.24		[70]
103	2-formyl-1-methylpyrrole	0.71		[70]
	**Sulfur compounds**			
104	Methanethiol	0.11–0.16	0.10–0.12	[10,47,49,67,68]
105	2-Propyl-thiophene	0.11		[70]
	**Phenolic compounds**			
106	2-Methoxyphenol (guaiacol)	0.02–9.12	0.01–0.04	[10,47,49,67,68,69,70]
107	4-Ethylguaiacol	1.81–4.85		[47,70]
108	4-Vinylguaiacol	3.24		[47]
109	4-Ethenyl-2-methoxyphenol	0.45		[69]
110	Phenol	1.00		[70]
111	4-Vinyl-2-methoxy-phenol	4.19		[70]
	**others**			
112	Pyridine	5.79–11.90		[69,70]
113	3-Ethyl-3-methyl maleic anhydride	0.09		[70]
114	Trimethyl oxazole	0.06		[70]
115	3-methyl 2(1*H*)-quinolinone	0.30		[70]
116	2-Methyl pyridine	0.03		[70]
117	3-Ethylpyridine	0.18		[70]
118	Linalool oxide	0.16		[70]
119	Linalool	0.08		[70]
120	*β*-Myrcene	0.06		[70]
121	*D*-Limonene	0.05		[70]

**Table 2:** The main odor-active compounds A summary of important aroma compounds identified in EC.

Key Odorants Identified in EC	Odor Description	
**ALDEHYDES**		
2-Methylpropanal	Grassy, fermented/Buttery–oily	[67,73,78,79,80]
2-Methylbutanal	Malty, fermented/Buttery–oily	[73,78,79,80]
3-Methylbutanal	Almond, fruity/Buttery–oily	[73,78,79,80]
Hexanal	Fruity	[67,79]
(*E*)-2-Nonenal	Buttery–oily	[78,80]
Acetaldehyde	Fruity	[67,79]
Benzeneacetaldehyde	Sweet–fruity	[78]
Propanal	Fruity	[67,79]
**KETONES**		
2,3-Pentanedione	Buttery–oily, caramel-like	[73,78,79,80]
2,3-Butanedione	Buttery–oily	[78,79,80]
(*E*)-*β*-Damascenone	Sweet–fruity	[78,80]
1-Octen-3-one	Mushroom-like	[78,80]
**ACIDS**		
2-Methylbutyric acid	Acidic	[80]
3-Methylbutyric acid	Acidic	[78,80]
**FURANES**		
2-((Methylthio)methyl)furan	Smoke roast	[78,80]
2-Furfurylthiol (2-furanmethanethiol)	Smoke roast	[78,80]
**SULFUR COMPOUNDS**		
Dimethyl trisulfide	-	[78,80]
Methanethiol	Freshness	[67,79]
3-(Methylthio)propionaldehyde	Soy sauce	[78,80]
3-Methyl-2-butene-1-thiol	Smoke roast	[78,80]
3-Mercapto-3-methylbutanol	Smoke roast	[78,80]
3-Mercapto-3-methylbutylformate	green–blackcurrant	[78,80]
**FURANONES**		
4-Hydroxy-2,5-dimethyl-3(2*H*)-furanone	Sweet–caramel	[80]
4,5-Dimethyl-3-hydroxy-2(5*H*)-furanone	Sweet–caramel	[78,80]
2-Hydroxy-3-methyl-2-cyclopenten-1-one	Sweet–caramel	[78]
2-Ethyl-4-hydroxy-5-methyl-3(2*H*)-furanone	Sweet–caramel	[78]
**PHENOLIC COMPUNDS**		
2-Methoxyphenol (guaiacol)	Phenolic, spicy	[67,73,78,79,80]
4-Ethyl-2-methoxyphenol (4-ethylguaiacol)	Phenolic	[78,80]
4-Ethenyl-2-methoxyphenol (4-vinylguaiacol)	Phenolic	[78,80]
**PYRAZINE**		
2-Ethylpyrazine	Earthy, musty	[73,79]
2-Ethyl-6-methylpyrazine	Earthy, musty/Earth, mould	[67,73,79]
2-Ethyl-3,5-dimethylpyrazine	Woody,papery/burned/nutty roast	[67,73,78,79,80]
2,3-Diethyl-5-methylpyrazine	Nutty roast	[78,80]
2-Methoxy-3- isopropylpyrazine	Green–earthy	[78,80]
2-Methoxy-3-(1- methylpropyl)pyrazine	Green–earthy	[78,80]
**TERPENE**		
Linalool	Sweet–fruity	[78,80]
**OTHERS**		
(3,4-Dihydro-2*H*-pyrrol-5-yl)-ethanone	Nutty roast	[78]
3-Methyl-1*H*-indole	Phenolic	[78]

**Table 3:** Main variables that influence the extraction process and EC flavor.

Variables	Constant Conditions	Chemical Analysis	Flavor Profile	Reference
*Time (s)*				
t1: 0–8 s t2: 9–16 s t3: 17–24 s	P (atm): 9 atm T (°C): 92 °C Grinding Grade: 6, 6.5, 7 (corresponding to fine, fine-coarse, and coarse	• Caffeine ↓ when the extraction time increases	• Compounds responsible for the aromatic profile ↓ when the ex-traction time increases. Their concentration is higher in samples with the finest grinding grade suggesting that by reducing the particle size, the extraction increases	[11]
Espresso Alba. t(s): 28.9 Espresso Classic t(s): 22 Espresso Intense. t(s): 23.5	For all capsules: T° and P: n.g	VOCs: maximum in-tensity 2–24 s. In the first 10s almost 95% of the VOCs are extracted More polar compounds are extracted faster.	• Not given	[92]
EC1: t(s): 28.7; T°(°C): 92;P (bar): 9 EC2:t(s): 24; T°(°C): 90; P: n.g EC3: t(s): 24; P(bar):19; T°(°C): n.g.	t (s): 24. T°, P: n.g PS: n.g.	Extraction efficiency per gram of coffee 3-CQA and 5-CQA: ↑EC3. ↓EC1 and EC2 Extracted acids: ↓EC1 and EC2. ↑EC3. Intensity aroma compounds: ↑EC3 ↓EC2 and EC1. Percentage fatty acids: ↑EC2 ↓ EC3 Extraction efficiency VOCs: ↑ intensity ECs. CQAs: ↑ECs.	ECs: ↑texture/body, strong roast, and bitter flavor, prolonged af-tertaste sensation. EC1 and EC2: ↑overall and roasty aroma in-tensity. EC1: fine, darker crema than EC2.	[3]
t1: 0–8, t2: 8–16, t3: 16–24, tf: 24.	t (s): 24. T°, P: n.g PS: n.g.	Increase time: ↓antioxidant capacity. t1 (0–8s): ↑70% antioxidant capacity, ~70% of 3-4-5CQA and ~50% of diCQAs extracted. t2 (8–16s): ~ 17% of 3-4-5CQA and ~30% diCQAs extracted. t3 (16–24s): ↓12% antioxidant capacity. ↓14% of 3-4-5CQA and ~20% diCQAs extracted.	Not given	[91]
*T (°C)*				
T°1 = 88 T°2 = 92 T°3 = 96 T°4 = 98	t(s): set up 21 P (bar): fixed 9	88 °C: ↑ 3-methylbutanal, 2-methylpropanal, 2- methylbutanal. 92 °C: ↑trigonelline, CQA. ↓ Pyrazines. ↑ Sulphur compounds, aldehydes, and ketones. 96 °C: ↓ trigonelline and CQA. ↑ pyrazines. 98 °C: ↓ trigonelline and CQA. ↑ hexanal	88 °C: ↑ odor, flavor, body, and overall ac-ceptability. 92 °C: ↑ freshness, fruity, malty, and buttery (positive notes), flavor, and overall acceptability.	[49]
T°1 = Up-drawn gradient (88–93). T°2 = Downdrawn gradient (93–88) T°3 = Fixed 90	t(s): 25. P (bar):9. PS (μm): 200–630^b^	T°1=↑TPC extraction, TS, 5-CQA T°2 = ↑total lipids, extraction yield to arabica washed coffees. T°3 = ↑caffeine and pH	T°1: balance, astringency, and bitterness. Good color of crema, well-balanced aroma intensity, body, and flavor. T°2 and T°3: ↓ foam index, viscosity, body, and level of pleasant odors. ↑ Bitterness and astringency.	[32]
T1 (°C): 90 T2 (°C): 100 T3 (°C): 110	P (atm): 12 Percolation time: 23-26 s	• Increasing temperature: ↑caffeine and foam index	Data not shown	[94]
*P (bar)*				
P1 = 7 P2 = 9 P3 = 11	t (s): set up 21. T°(°C): fixed 92. PS: fine grinding ^a^	7 bar: ↓lipids and CQAs. ↓Methanethiol acetaldehyde, propanal, 3-methylbutanal, 2,3-butanedione. 9 bar: ↑ lipids, CQAs, ↑odor compounds. ↑Methanethiol and propanal. 11 bar: ↓ lipids and CQAs, ↑2-methylbutanal, 3- methylbutanal, 2-ethyl-3,5 dimethylpyrazine.	7 bar: ↑acrid, straw, malty, cereal notes. 9 bar: ↑ key odorants related to freshness, fruity, malty, and buttery. 11 bar: ↑ bitterness, astringency, odor and aftertaste intensity, notes cereal/malty notes, burnt/roasty. ↓ Overall acceptability.	[10]
*PS (μm)* ^b^				
Very fine (VF) = 450 Fine (F) = 550 Coarse (C) = 550–600	t (s): Set up 2 T° (°C): Fixed 92 P (bar): Fixed 9	VF: ↑trigonelline, lipids, caffeine, and CQAs. ↑2- methylpropanal, 2-methylbutanal, 3-methylbutanal, 2,3- butanedione and 2,3-pentanedione. F: trigonelline, lipids, caffeine, and CQAs. ↑ 2- methylpropanal, 2 methylbutanal, 3-methylbutanal. C: ↓trigonelline, lipids, caffeine, and CQAs.	VF: slightly over-extracted, presence of woody/papery, fermented, burnt/roasty notes. F: ↑body, woody/papery, fermented, burnt/roasty notes. C: ↓ development of aroma and flavor. Presence of the acrid, burnt and rubbery notes.	[49]
*P (bar) + T (°C)*				
ECA: settable P (bar) = 7, 9, 11 T° (°C) = 88, 92, 98. ECB: unsettable P (bar) = ~2–9 T° (°C) = ~87-98	t (s): fixed 25. PS: n.g (fine grindinga)	ECA: ↑ aroma intensity (92 °C/9 bar). ↑ Positive key odorants the final fractions (21–25s). ECB: ↑ proteins, lipids, and positive key odor-ants in the first frac-tions (0–10 s).	ECA: ↑ positive con-tribution of the key odorants at 92 °C/9 bar ↓ negative flavor notes. ↑ aroma inten-sity ECB: ↓ positive odor-ants in the intermedi-ate and last fractions.	[40]
P, T°1: 9/92. P, T°2: 7/92. P, T°3: 11/92. P, T°4: 9/82. P, T°5: 9/96	P, T°4: 9/82. P, T°5: 9/96	Increasing T°: ↑VOCs intensity, especially t > 14 s ↑ solubility, ↑extraction. 11 bar: ↑ VOCs over the entire extraction time than at 7 bar. 7 and 9 bar: No differ-ences in VOCs families. ↑P and ↑T°: ↑VOCs extraction.	• Least polar com-pounds are the most affected, impacting the aroma balance in the last stage of the extraction and the cup.	[95]
*P (bar) + T (s)*				
EC (Es-presso Coffee): P (bar): 9; t(s): 27 ECF (Espresso Coffee Firenze): P (bar): 20; t (s): 70 ECS (Specialty Espresso Coffee): P (bar): 9 T (s): 26	• T° (°C): 92–93 °C	Caffeine content: ↑ EC, ECS; ↓ ECF Chlorogenic acids content: ↑ EC, ECS; ↓ ECF	Not given	[98]

EC: Espresso coffee; t: time; T°: Temperature; P: pressure, PS: Particle sizes; CQAs: caffeoylquinic acids. TPC: total phenolic compounds VOCs: volatile organic compounds. An upward arrow (↑) refers to an increase or high values within conditions evaluated. A downward arrow (↓) refers to a decrease or low values within conditions evaluated. ^a^ designates the level of grinding or particle sizes that were reported, but the method used for measuring is not reported; ^b^ Particle size characterization was performed by analysis with sieves using a certain amount of roasted and ground coffee (usually, 100 g)**.**

**Table 4:** Major analytical techniques used for the determination of volatile compounds in Espresso Coffee.

	Sampling	Analyte Separation	Detecting System	References
**GC-based method**	1. Solvent-assisted extraction	1. Monodimentional (ZB-FFAP, HP-WAX, DB-WAX, DB-5)	1. Mass Spectrometer (GC-MS) - Quadrupole MS - Q-ToF	[8,10,11,40,67,69,75,76,104,105,106]
	2. Static Headspace (SHS)			
	3. Headspace solid-phase microextraction (HS-SPME)	2. Multidimentional GC x GC (DB-5 x Supelcowax 10)	2. Flame Ionization Detector (GC-FID)	[107]
	4. Dynamic HS			
	5. Headspace sorptive extraction (HSSE) and stir bar sorptive extraction (SBSE)		3. Olfactometry detector (GC-O) - Aroma Extract Dilution Analysis (AEDA) - Odor activity values (OAVs)Odor spectrum values (OSV)	[25,79]
**Proton Transfer Reaction-MS (PTR-MS)**	Direct Injection of VOCs from HS	Not applicable	PTR-ToF-MS	[92,108,109]
**Electronic Nose (EN) sensing**	HS	Not applicable	Metal-oxide Sensors (MOS)	[11,76]

## Data Availability

Not applicable.
